# Delayed Arteriovenous Fistula Maturation Associated With an Ipsilateral Transvenous Implantable Cardioverter Defibrillator (ICD) Lead and Suspected Functional Hemodynamic Obstruction

**DOI:** 10.7759/cureus.113325

**Published:** 2026-07-24

**Authors:** Vickram Reddy, Vivek Reddy, Syed Hussain

**Affiliations:** 1 Internal Medicine, Riverside Medical Center, Kankakee, USA; 2 Electrophysiology, Riverside Medical Center, Kankakee, USA; 3 Vascular Surgery, Riverside Medical Center, Kankakee, USA

**Keywords:** arteriovenous fistula, central venous stenosis, functional obstruction, hemodialysis access, hemodynamic perturbation, maturation failure, subcutaneous icd, transvenous icd

## Abstract

Arteriovenous fistula (AVF) maturation failure is a possible barrier to establishing permanent hemodialysis access. While overt stenosis is the most commonly cited cause, a substantial proportion of non-maturing fistulas lack angiographically apparent lesions, suggesting that subclinical hemodynamic and geometric factors play a critical role. This case describes a 65-year-old man with end-stage renal disease on hemodialysis, coronary artery disease status post right coronary artery (RCA) stent placement, ischemic cardiomyopathy (left ventricular ejection fraction (LVEF) 40-45%), and a history of sustained ventricular tachycardia requiring single-chamber transvenous implantable cardioverter defibrillator (ICD) placement, who underwent left upper extremity AVF creation ipsilateral to the device. Despite serial fistulograms demonstrating patent central veins without discrete stenosis, the fistula failed to mature over five months. The clinical course and sequential interventions--coil embolization of accessory venous branches, transvenous ICD extraction with subcutaneous ICD placement, and continued surveillance--were followed by clinical improvement and a patent AVF that was cleared for hemodialysis use. The temporal association between lead extraction and subsequent clinical improvement and clearance of the AVF for hemodialysis use is consistent with the hypothesis that the ipsilateral transvenous ICD lead may have contributed to delayed maturation through subclinical flow perturbation rather than overt mechanical stenosis. This case highlights the importance of considering ICD platform selection as a modifiable factor in dialysis access planning and illustrates the value of transitioning from transvenous to subcutaneous ICD to preserve vascular access in dialysis-dependent patients.

## Introduction

The arteriovenous fistula (AVF) is the preferred vascular access for chronic hemodialysis, yet maturation failure remains a significant barrier, with up to 60% of newly created AVFs failing to mature in some cohorts [1]. Although the traditional "rule of sixes" (flow ≥600 mL/min and vein diameter ≥6 mm) has historically been used to assess AVF maturation, the 2019 Kidney Disease Outcomes Quality Initiative (KDOQI) guidelines emphasize clinical usability and individualized assessment rather than fixed ultrasound thresholds alone [1]. While overt stenosis is the most commonly recognized cause of failure to mature (FTM), a substantial proportion of non-maturing fistulas lack angiographically apparent lesions [1]. This observation suggests that disturbed flow dynamics and vascular geometry may contribute to maturation failure despite a patent venous lumen. Functional hemodynamic obstruction refers to impaired blood flow resulting from alterations in flow velocity and wall shear stress without an anatomically visible stenosis. Such disturbances may interfere with the outward vascular remodeling required for successful AVF maturation [2,3].

The intersection of cardiac implantable electronic devices (CIEDs) and hemodialysis access adds further complexity. Transvenous leads are associated with central venous stenosis in 11-36% of patients, with symptomatic stenosis occurring in up to 71% of patients when the AVF and lead are ipsilateral [3]. Accordingly, current recommendations favor contralateral placement whenever feasible [3].

To our knowledge, reports describing delayed AVF maturation that improved following transvenous lead extraction despite the absence of angiographically apparent central venous stenosis remain limited. This case explores the hypothesis that an ipsilateral transvenous implantable cardioverter defibrillator (ICD) lead may have contributed to delayed AVF maturation through functional hemodynamic disturbance rather than overt mechanical obstruction and highlights the potential implications of ICD platform selection for preservation of dialysis vascular access.

## Case presentation

A 65-year-old right-hand-dominant man with end-stage renal disease (ESRD) on hemodialysis presented for vascular access planning. His medical history was significant for coronary artery disease status post right coronary artery (RCA) stent placement two years prior, subsequently found to be occluded within the stent on catheterization approximately one year later with left-to-right collaterals. He carried a diagnosis of ischemic cardiomyopathy with a left ventricular ejection fraction (LVEF) of 40-45% and multivessel coronary artery disease. Additional comorbidities included severe chronic obstructive pulmonary disease (COPD) with chronic hypoxemic respiratory failure requiring home oxygen.

Approximately three months prior to AVF creation, the patient developed sustained ventricular tachycardia and acute kidney injury requiring initiation of hemodialysis. A single-chamber transvenous ICD was implanted approximately two months prior to AVF creation via the left subclavian vein. A right internal jugular vein hemodialysis catheter was placed during the same hospitalization.

Preoperative venous mapping, performed approximately two weeks prior to AVF creation, demonstrated a left basilic vein of at least 6 mm above the elbow--superior to the right-sided options (right basilic 2 mm, right cephalic 3 mm). The left cephalic vein measured approximately 4-5 mm. Given the inadequacy of right-sided venous conduits, a left upper extremity AVF was created between the median cubital vein and brachial artery on Day 1, ipsilateral to the transvenous ICD.

**Figure 1:**
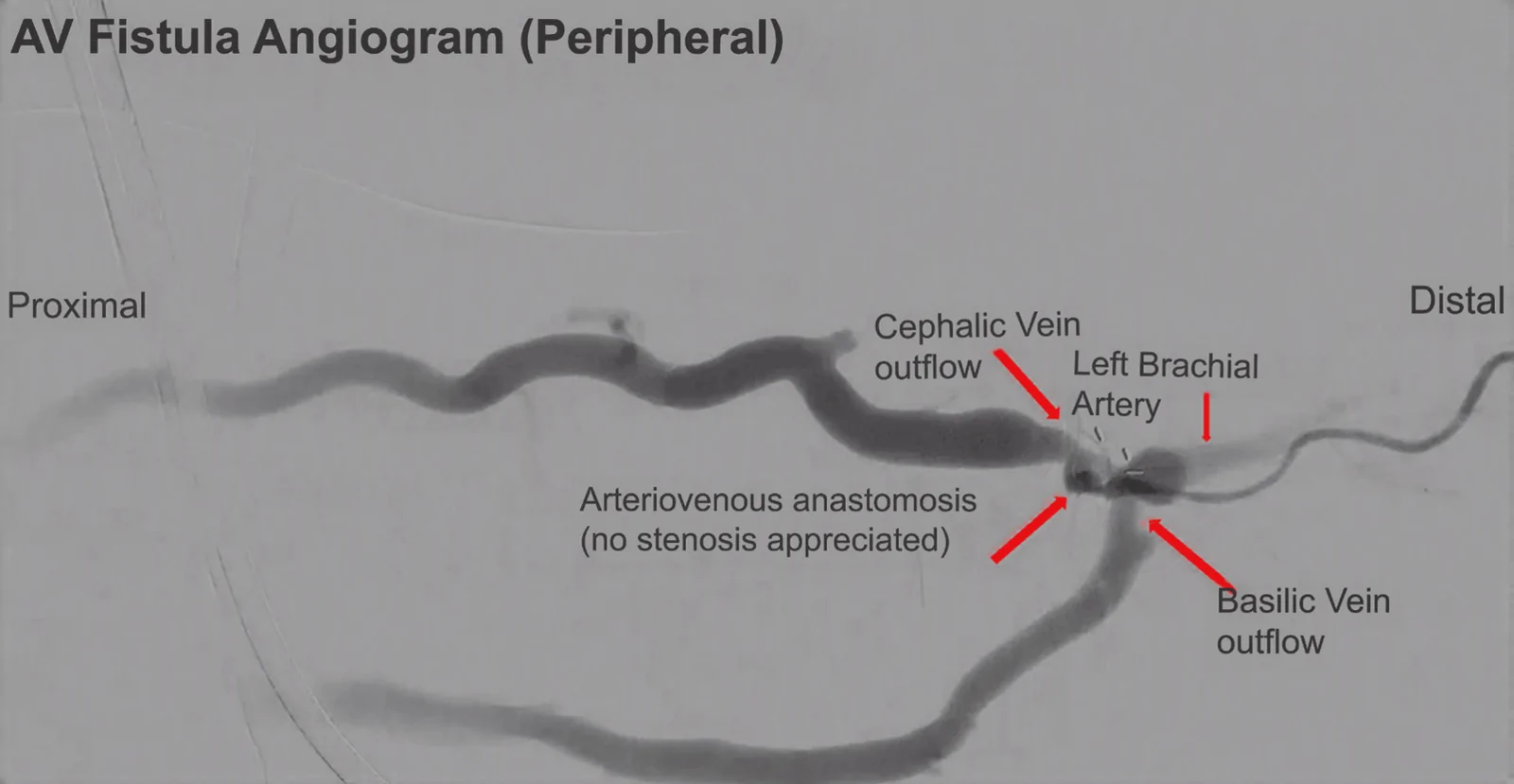
Day 89, AV fistula angiogram (peripheral) Peripheral fistulogram obtained at Month 3 (Day 89) demonstrating the arteriovenous (AV) anastomosis between the median cubital vein and brachial artery with outflow through the cephalic and basilic veins. The left brachial artery is visualized. No hemodynamically significant stenosis is identified in the peripheral venous outflow tract despite clinical immaturity of the fistula.

**Figure 2:**
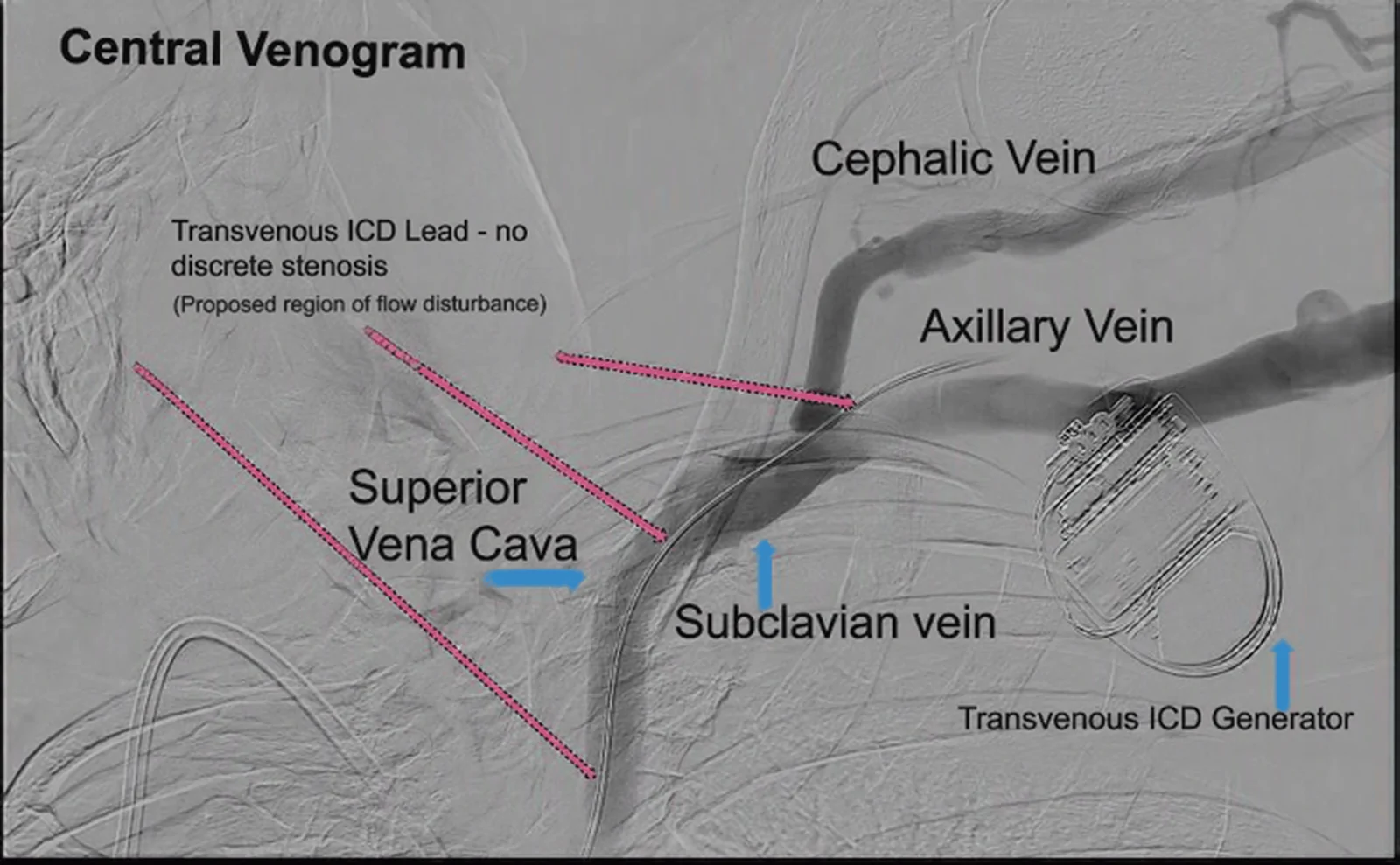
Day 89, AV fistula angiogram (central) Central venogram obtained at Month 3 (Day 89) showing the left cephalic vein, axillary vein, and subclavian vein draining into the superior vena cava (SVC). The transvenous implantable cardioverter defibrillator (ICD) lead and generator are visualized traversing the left subclavian vein. Despite the presence of the ipsilateral transvenous lead, no discrete central venous stenosis is identified. The lead-vein interface is annotated as the proposed region of hemodynamic disturbance; this mechanism was not directly measured.

At Day 89, the patient was evaluated for AVF maturation. On examination, the fistula had not matured, and the patient reported worsening left upper extremity swelling. Duplex ultrasound demonstrated a patent left upper extremity AVF with a maximum volume flow of 589 mL/min. The AVF was located 3.8-5.8 mm beneath the skin surface, with a peak systolic velocity of 344.2 cm/s at the anastomosis. Subcutaneous edema was present near the anastomosis without evidence of perigraft fluid or hematoma. Fistulography demonstrated a patent arteriovenous anastomosis with outflow through the cephalic and basilic veins without hemodynamically significant stenosis (Figure 1). Central venography confirmed widely patent axillary, subclavian, brachiocephalic, and superior vena cava segments despite the presence of the ipsilateral transvenous ICD lead, with no discrete central venous stenosis identified (Figure 2). Given the absence of a correctable stenotic lesion, continued surveillance was recommended with consideration of accessory vein embolization if maturation did not progress.

At Day 201, the fistula remained immature despite continued observation. Repeat fistulography demonstrated widely patent central and peripheral venous outflow without evidence of hemodynamically significant stenosis. Clinical examination revealed a strong thrill near the antecubital fossa but minimal palpable thrill in the mid-upper arm. Fistulography and ultrasound identified two large accessory cephalic vein branches diverting flow from the primary outflow tract. Coil embolization of both accessory branches was performed, and the final fistulogram demonstrated improved flow through the cephalic vein with minimal residual flow through the embolized branches.

At Day 265, the patient returned for post-embolization follow-up. There was minimal improvement in left upper-extremity swelling, and the fistula remained unsuitable for dialysis use. Given the persistent failure to mature despite patent central veins and successful accessory vein embolization, the ipsilateral transvenous ICD lead was identified as a potential contributing factor. The decision was made to proceed with transvenous ICD extraction and transition to a subcutaneous ICD (S-ICD) to eliminate the intravascular lead as a source of hemodynamic perturbation.

At Day 299, the transvenous ICD was extracted, and a subcutaneous ICD was placed. The procedure was complicated by a small post-procedural hematoma at the abdominal generator site, which resolved with binder compression. The patient’s right-sided tunneled dialysis catheter was used for ongoing hemodialysis access during the interval of continued AVF maturation.

**Figure 3:**
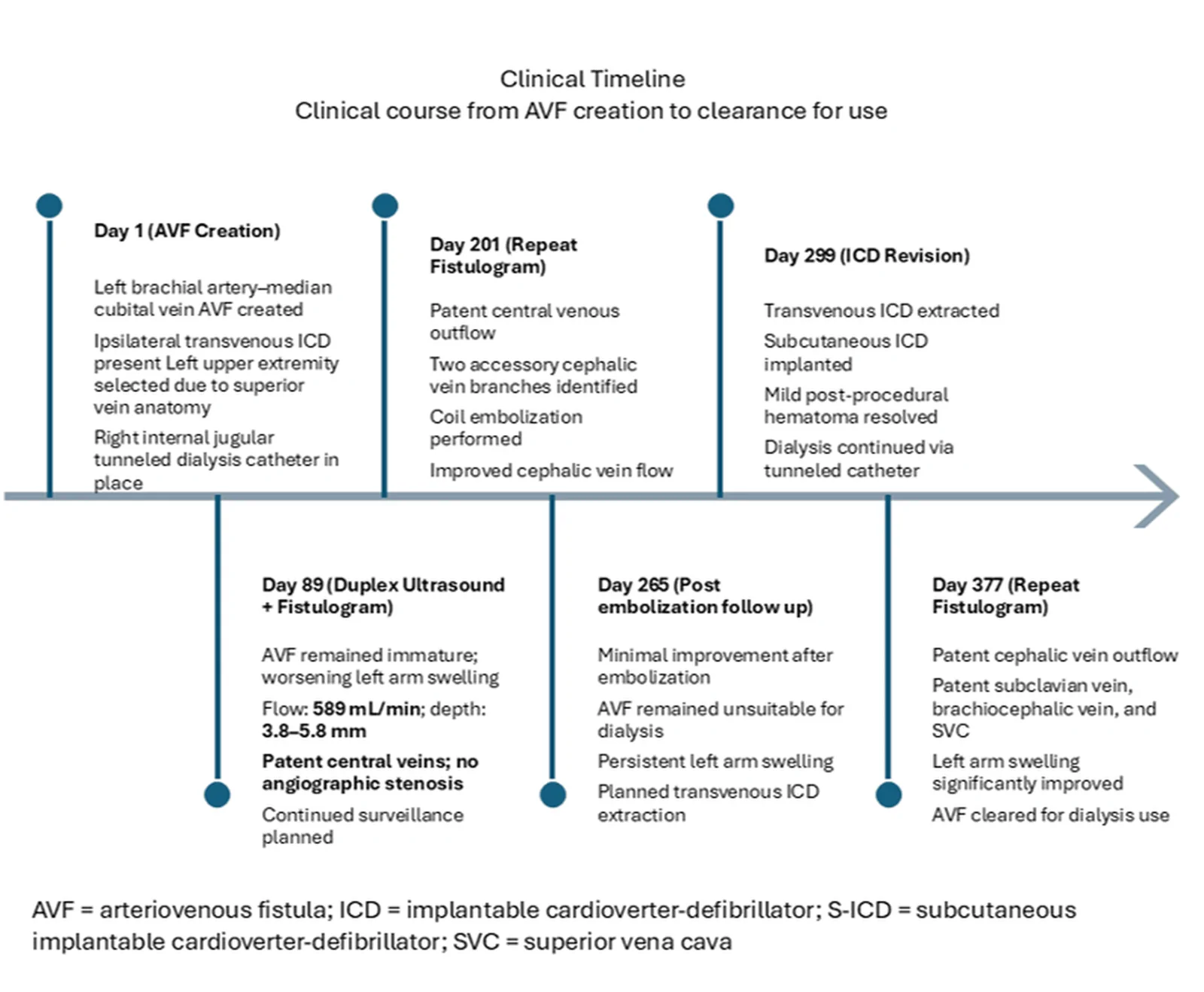
Clinical timeline from AVF creation to clearance for hemodialysis use Clinical timeline illustrating the patient's course from AVF creation (Day 1) to clearance for hemodialysis use (Day 377). Key milestones include duplex ultrasound and fistulography demonstrating patent central veins without angiographic stenosis (Day 89), accessory vein coil embolization (Day 201), persistent non-maturation despite intervention (Day 265), transvenous ICD extraction with subcutaneous ICD implantation (Day 299), and repeat fistulography demonstrating patent central venous outflow with subsequent clearance of the AVF for dialysis use (Day 377).

At Day 377, the patient reported marked improvement in left upper-extremity swelling. Repeat fistulography demonstrated a patent cephalic vein outflow tract throughout the distal, mid, and proximal segments, with widely patent venous confluence, subclavian vein, brachiocephalic vein, and superior vena cava. Only small residual proximal venous branches remained, and the AVF was cleared for hemodialysis use. Subsequent successful cannulation and sustained functional use were not available in the record reviewed. The clinical course is depicted in Figure 3.

## Discussion

This case illustrates the multifactorial nature of AVF maturation failure in a patient whose serial fistulograms consistently demonstrated patent central veins without discrete stenosis. Multiple factors likely contributed to impaired maturation, including reduced cardiac output from ischemic cardiomyopathy (LVEF 40-45%), accessory venous branches diverting flow from the main outflow tract, vascular geometry at the anastomotic site, and the uremic milieu of ESRD, all of which are well-established contributors to AVF dysfunction [1,2]. However, the clinical course raises the possibility that the ipsilateral transvenous ICD lead may have served as a potentially important and modifiable contributor, not through overt mechanical obstruction or angiographic stenosis, but through subclinical hemodynamic perturbation that impaired the flow dynamics necessary for fistula maturation. The improvement in upper extremity swelling and subsequent clearance of the fistula for hemodialysis use following lead extraction is consistent with, but does not establish, this interpretation.

Proposed role of the transvenous lead in functional hemodynamic disturbance

The central paradox of this case--a non-maturing AVF with patent central veins--may be explained by the concept of functional obstruction. Transvenous leads do not need to produce angiographic stenosis to impair venous hemodynamics. Computational analyses have demonstrated that leads create focal pockets of low velocity and shear stress between the vein wall and the lead, upregulating inflammatory markers and promoting a prothrombotic milieu even in the absence of visible narrowing [4]. Early after lead insertion, endothelial damage and flow perturbation trigger an inflammatory cascade mediated by neutrophils, macrophages, and foreign body giant cells, with circulating procoagulant factors increasing soon after placement [4]. Over time, fibrin deposition on the lead surface incorporates into the intima, with smooth muscle cell infiltration and eventual collagen encapsulation that further reduces the functional venous diameter without necessarily producing a discrete stenotic lesion [4].

This distinction between functional and mechanical obstruction is clinically important. Standard two-dimensional (2D) fistulography evaluates luminal patency but cannot detect the hemodynamic disturbances created by a lead occupying the venous lumen. A transvenous lead may reduce the effective cross-sectional area available for flow and contribute to disturbed flow at the lead-wall interface, creating conditions analogous to the disturbed flow patterns that drive neointimal hyperplasia at swing points in AVFs [3]. Although the traditional rule of sixes uses a flow of at least 600 mL/min and a vein diameter of at least 6 mm as reference thresholds, the 2019 KDOQI guidance emphasizes individualized clinical usability rather than fixed ultrasound criteria alone. Even modest hemodynamic disturbance may interfere with the outward remodeling required for maturation [1].

The epidemiologic data are consistent with this proposed mechanism. Symptomatic central vein stenosis was reported in up to 71% in selected cohorts of hemodialysis patients when the AVF and transvenous lead were positioned ipsilaterally, at a mean of 12.6 months after AVF creation [3]. In a large venographic study of over 3,000 CIED carriers, proper AVF function was uncertain in nearly 50% of patients ipsilaterally versus only 2% contralaterally [5]. The time to first intervention is notably shorter with ipsilateral access compared to contralateral placement [3,6]. These data suggest that the lead's hemodynamic footprint--its cumulative effect on flow dynamics, endothelial activation, and venous remodeling--may be substantial even when conventional imaging appears reassuring.

Compounding factors

While the transvenous lead may have represented a potentially important contributing factor, several non-modifiable factors likely compounded the hemodynamic insult. Pre-existing systolic dysfunction is among the most powerful predictors of AVF maturation failure; patients with systolic dysfunction are more than five times less likely to achieve maturation by one year [7]. LVEF is independently associated with unassisted maturation, and this patient's LVEF of 40-45%, combined with extensive multivessel coronary artery disease, placed him in a high-risk category based on cardiac output alone [7,8]. Reduced cardiac output limits the hemodynamic drive--the sustained high-flow, high-shear environment--necessary for the compensatory vascular remodeling that underlies successful maturation [1,8].

Accessory venous branches, identified and embolized with coils during the clinical course, diverted flow from the main outflow tract. While these branches do not cause stenosis of the main channel, they reduce the effective flow volume through the maturing vein, further diminishing the shear-stress stimulus for outward remodeling [1]. The vascular geometry at the anastomotic site--appreciated clinically as kinking--is consistent with a swing-point mechanism, where sharp angulation creates nonlaminar flow and decreased wall shear stress that can trigger neointimal hyperplasia [3]. Computational fluid dynamics studies have consistently linked disturbed flow patterns, including low wall shear stress and high oscillatory shear index, to regions prone to neointimal hyperplasia and stenosis [3,9]. Importantly, these hemodynamic disturbances are invisible on standard 2D fistulography.

The convergence of these factors--a proposed lead-related hemodynamic disturbance superimposed on reduced cardiac output, accessory branch steal, unfavorable vascular geometry, and the uremic milieu--may explain why this fistula failed to mature despite apparently patent central veins. Each factor alone may have been insufficient to prevent maturation, but their combination created a hemodynamic environment inhospitable to the outward remodeling process.

Clinical implications: the case for s-ICD in dialysis patients

The clinical improvement and subsequent clearance of the AVF for hemodialysis use after transvenous lead extraction and S-ICD placement may have important implications for providers managing the intersection of CIED therapy and hemodialysis access. When a transvenous ICD is identified as a potential contributor to impaired AVF maturation, the clinical decision is not simply whether to relocate the AVF to a contralateral site, which may not be feasible given limited vascular options, but whether to transition the ICD platform itself to one that leaves the venous system entirely untouched.

The S-ICD and transvenous ICD differ fundamentally in their relationship to the vasculature and their therapeutic capabilities. The transvenous ICD places leads through the subclavian vein into the cardiac chambers, enabling bradycardia pacing, antitachycardia pacing (ATP), and cardiac resynchronization therapy (CRT) in addition to defibrillation. The S-ICD, in contrast, is implanted entirely in the extrathoracic subcutaneous space with a lead positioned superficial to the sternum, providing defibrillation without any intravascular components. This avoids risks directly attributable to intravascular leads, including lead-related venous obstruction, endovascular infection, central venous stenosis, and endovascular infection, but at the cost of being unable to provide bradycardia pacing (beyond brief post-shock pacing), ATP, or CRT.

The inability to deliver ATP is the most clinically significant limitation. In the PRAETORIAN trial, the first ATP attempt successfully terminated 46% of monomorphic ventricular tachycardias, but accelerated the arrhythmia in 9.4% of cases [10]. Patients in the S-ICD group were more likely to receive an ICD shock (19.2% vs. 11.5% at 48 months; P=0.02), though the total number of appropriate shocks was not significantly different between groups, and first shock efficacy was comparable (93.8% vs. 91.6%) [10]. The PRAETORIAN-XL extension at 87.5 months of follow-up demonstrated that transvenous ICD patients had significantly more major complications and lead-related complications than S-ICD patients, and the as-treated analysis showed significantly more overall complications with transvenous ICD (hazard ratio (HR) 0.64; P=0.047) [11]. These data led the investigators to conclude that the S-ICD should be considered for all patients without a pacing indication who are evaluated for ICD therapy [11].

For dialysis patients specifically, the rationale for S-ICD is particularly compelling. The 2017 AHA/ACC/HRS guidelines provide a Class I recommendation that patients who meet ICD criteria and have inadequate vascular access or are at high risk for infection, including those with ESRD, should receive an S-ICD when pacing is neither needed nor anticipated [12]. The 2024 AHA Scientific Statement on CIED infections similarly recommends consideration of S-ICD in patients at high risk of infection, explicitly including hemodialysis-dependent kidney disease [13]. The ACC/AHA/HRS 2025 Appropriate Use Criteria note that due to the increased risk for endovascular infection in patients on chronic dialysis, the S-ICD is now often considered as an alternative to transvenous ICD systems [14]. Nationally, the proportion of S-ICDs among dialysis patients increased from 10% in 2012 to 69% in 2018, reflecting rapid adoption of this strategy [15].

Should S-ICD be used empirically in dialysis patients?

Current guidelines do not mandate S-ICD over transvenous ICD in all dialysis patients, but the trajectory of evidence and practice patterns suggests this may be an area of evolving consensus. The existing Class I recommendation is framed around patients with "inadequate vascular access or high risk for infection," and ESRD patients on hemodialysis meet both criteria. The JACC State-of-the-Art Review on CIEDs in chronic kidney disease (CKD) patients recommends that AVF and transvenous leads be positioned contralaterally when possible but acknowledges that limited vascular sites and frequent AVF revisions make this challenging [3]. When contralateral placement is not feasible, as in this patient, whose right-sided venous options were inadequate, the choice becomes between accepting the potential vascular-access consequences of ipsilateral transvenous lead placement or transitioning to a platform that avoids the venous system entirely.

This case raises the question of whether empiric S-ICD placement should be considered at the time of initial ICD implantation in dialysis patients, rather than reserving it for situations where transvenous leads have been implicated in complications. The rationale would be proactive preservation of vascular access--a finite and life-sustaining resource in dialysis-dependent patients--rather than reactive management after maturation failure has occurred. The primary barrier to this approach is the S-ICD's inability to provide ATP or bradycardia pacing, which limits its use in patients with concomitant bradycardia or frequently recurring monomorphic ventricular tachycardia (VT) amenable to ATP. Notably, renal dysfunction is associated with conversion of S-ICD to transvenous ICD (odds ratio (OR) 2.67; P=0.008) due to subsequent need for pacing or CRT [3]. The emerging extravascular ICD (EV-ICD), which places a lead in the substernal space and can provide both ATP and pause-prevention pacing without intravascular components, may eventually bridge this gap, though further data in dialysis populations are needed [16].

For the broader multidisciplinary team--vascular surgeons, nephrologists, interventional radiologists, and electrophysiologists--this case underscores the importance of early, coordinated planning. When a dialysis patient requires ICD therapy, the decision about device platform should incorporate not only the arrhythmia substrate and pacing needs but also the current and anticipated vascular access requirements. If the patient does not require bradycardia pacing, ATP, or CRT, the S-ICD offers a strategy that preserves the venous system for hemodialysis access without compromising defibrillation efficacy.

Limitations

This report has several limitations. As a single observational case, it cannot establish causality between the ipsilateral transvenous ICD lead and delayed AVF maturation, and its findings may not be generalizable to other patients with cardiac implantable electronic devices and hemodialysis access. Although improvement followed lead extraction, delayed natural maturation, the preceding accessory-vein embolization, and other patient-specific factors may also have contributed to the observed course. Serial duplex-derived access-flow measurements, outflow-vein diameter measurements, venous pressure measurements, quantitative assessment of upper-extremity swelling, and direct computational or physiologic evaluation of flow disturbance were unavailable. In addition, although the AVF was cleared for hemodialysis use, subsequent successful cannulation, sustained functional use, dialysis adequacy, and tunneled catheter removal were not confirmed. Accordingly, the proposed mechanism of lead-related functional hemodynamic disturbance should be considered hypothesis-generating and warrants prospective investigation.

## Conclusions

This case suggests that delayed AVF maturation may occur in the absence of angiographically apparent central venous stenosis in patients with ipsilateral transvenous ICD leads. The temporal association between lead extraction and subsequent clinical improvement raises the possibility that lead-related flow disturbance may have contributed alongside reduced cardiac function, accessory venous branches, vascular geometry, and the uremic milieu. Because direct hemodynamic measurements were unavailable and delayed natural maturation or the effect of prior accessory-vein embolization cannot be excluded, this observation should be considered hypothesis-generating. Prospective studies incorporating serial access-flow measurements, objective hemodynamic assessment, and clinical usability outcomes are needed to determine whether lead-related flow disturbance influences AVF maturation and should inform ICD platform selection in dialysis-dependent patients.
